# Strategies for Coordination of a Serosurvey in Parallel with an Immunization Coverage Survey

**DOI:** 10.4269/ajtmh.15-0198

**Published:** 2015-08-05

**Authors:** Mark A. Travassos, Berhane Beyene, Zenaw Adam, James D. Campbell, Nigisti Mulholland, Seydou S. Diarra, Tassew Kassa, Lisa Oot, Jenny Sequeira, Mardi Reymann, William C. Blackwelder, Marcela F. Pasetti, Samba O. Sow, Robert Steinglass, Amha Kebede, Myron M. Levine

**Affiliations:** Center for Vaccine Development, University of Maryland School of Medicine, Baltimore, Maryland; Ethiopian Public Health Institute, Addis Ababa, Ethiopia; JSI Research and Training Institute Inc., Arlington, Virginia; Department of Social Work, The Women's Hospital, Melbourne, Australia; Centre pour le Développement des Vaccins, Bamako, Mali

## Abstract

A community-based immunization coverage survey is the standard way to estimate effective vaccination delivery to a target population in a region. Accompanying serosurveys can provide objective measures of protective immunity against vaccine-preventable diseases but pose considerable challenges with respect to specimen collection and preservation and community compliance. We performed serosurveys coupled to immunization coverage surveys in three administrative districts (woredas) in rural Ethiopia. Critical to the success of this effort were serosurvey equipment and supplies, team composition, and tight coordination with the coverage survey. Application of these techniques to future studies may foster more widespread use of serosurveys to derive more objective assessments of vaccine-derived seroprotection and monitor and compare the performance of immunization services in different districts of a country.

## Introduction

Immunization services in developing countries administer multiple vaccines to children and pregnant women through routine immunization schedules that follow Expanded Program on Immunization (EPI) guidelines and through supplemental immunization activities that include periodic mass campaigns. Estimating vaccination coverage in a region provides governmental and international partner agencies valuable information on the effectiveness of these services. One means of generating such estimates is to perform immunization coverage surveys implemented according to World Health Organization (WHO) guidelines.^1^ Such surveys rely on family-held vaccination records that may be incomplete or missing and also on parental recall, which may be inaccurate. In contrast, a serosurvey that measures antibodies produced in response to vaccination provides an objective measure of immunization coverage and the induction of protective immunity. However, serosurveys also pose notable challenges, including the need to obtain community and individual family participation and difficulties in collecting venous blood samples from infants and toddlers in remote conditions and maintaining the integrity of specimens with a cold chain. Effective strategies and tactics for implementing a serosurvey in conjunction with a coverage survey, particularly in remote conditions, have not previously been described. Here, we describe the performance of a simultaneous coordinated immunization coverage survey and serosurvey in three rural districts in Ethiopia in 2013, focusing on the techniques that enabled successful execution of this challenging endeavor.

## Materials and Methods

**Figure 1.**
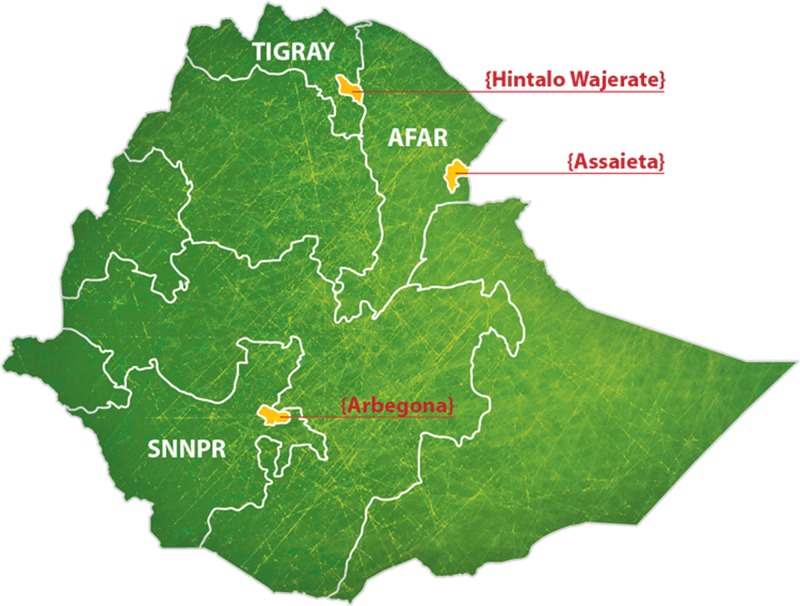
Map of Ethiopia indicating the location of each of the three woredas surveyed. This included Hintalo Wajerate woreda, Tigray Region; Arbegona woreda, Southern Nations, Nationalities, and Peoples' Region (SNNPR); and Assaieta woreda, Afar Region.

As part of an Ethiopian Ministry of Health project, children in each of three rural administrative districts (woredas) were selected to participate in immunization coverage surveys and accompanying serosurveys during February to April, 2013, dates chosen so that navigability would not be limited by seasonal rains. This included 400 children in each woreda, a sample size reflecting the sampling capacity of the serosurvey (*N* = 1,200 total). Selected regions included Hintalo Wajerate woreda, Tigray Region; Arbegona woreda, Southern Nations, Nationalities, and Peoples' Region (SNNPR); and Assaieta woreda, Afar Region (Figure 1

). Each woreda was selected by Ethiopian Regional Health Bureau and JSI Research and Training Institute Inc. (JSI) partners to inform the Federal Ministry of Health (FMOH) evidence-based decision on whether and how to pursue nationwide universal child immunization as part of the Universal Immunization through Improving Family Health Services Project (UI-FHS). These woredas were chosen given that nearly 90% of Ethiopia's population resides in similar rural regions.

The serosurvey protocol was approved by the Institutional Review Board of the University of Maryland, Baltimore, and the Ethiopian National Research Ethics Review Committee. Written informed consent was obtained from the parents of each child enrolled in the serosurvey. Participation in the immunization coverage survey, a routine FMOH public health endeavor, did not require written informed consent.

### Statistical sampling.

To obtain the sample populations, a total of 102 clusters were randomly selected from the most recent list of enumeration areas (EAs) from the Central Statistics Authority (CSA). The number of infants and children to be surveyed in each EA was then determined on the basis of probability sampling proportionate to size.^2^ Finally, a list of households with target age infants and children was created within the EA, and households were selected for the survey by systematic random sampling. With an additional 5% adjustment for expected nonresponse, a total of 2,080 households (700 each for Arbegona and Hintalo and 680 for Assaieta) were included. The target census for the coverage survey in each woreda was 300 toddlers 12–23 months of age and 100 infants aged 6–8 months, with typically 9 toddlers and 3 infants selected per cluster. The enrollment target for the serosurvey was 60% of the coverage survey enrollment.

### Immunization coverage survey.

#### Coverage survey team composition.

The immunization coverage survey in each woreda was conducted by an experienced team from Matrix Health and Development Solutions (Addis Ababa, Ethiopia). A coverage survey team included two enumerators. There was one local guide and one supervisor per two teams. Before the start of work in each woreda, enumerators and supervisors were recruited and given training on EPI essentials, survey tools (including use of a Global Positioning System [GPS] navigation device), and ethics with role play exercises and actual field practice. Training also included a mock exercise to determine the optimum number of households and time needed to administer questionnaires and collect blood specimens.

The coverage survey was executed following the WHO immunization coverage cluster survey reference manual,^1^ with the following differences:

1. All households surveyed were randomly selected, not selected based on proximity to the first household surveyed.
2. Data collection forms were tailored to the needs of the coverage survey and included an assessment of caregiver immunization knowledge, attitudes, and practices.

The coverage survey team initially visited each cluster, canvassing all homes for children either 6–8 months old or 12–23 months old. For households selected for the survey, in addition to recording data from immunization cards and completing the questionnaires for the coverage survey, the team recorded the latitude, longitude, and altitude of each household with a GPS navigation device. From a list of eligible children for each cluster, the team randomly selected nine children of 12–23 months old and three children of 6–8 months old. The immunization coverage survey team provided each selected household with the time, date, and a location where they would meet the serosurvey team (typically the same or following day) so that specimens could be collected from the infant or toddler; they were also given a referral slip to bring to aid identification. The coverage survey team also verified immunization records of children at the local health facility in the event of parents verbally reporting vaccination but not being able to produce the child's vaccination card.

### Serosurvey.

#### Core serosurvey group composition.

The serosurvey group was based at a local health facility, where equipment and supplies were stored and serum samples were processed, aliquoted, and kept in cold storage. Two identically structured serosurvey groups worked simultaneously so that subjects could be surveyed from two clusters at once. Teams met subjects at the central meeting location provided by the coverage survey team, which was typically a health post, school, or community center. The responsibilities of each team member overlapped so that a member could provide backup as needed (Table 1).

##### Team leader.

The team leader was a physician or nurse with field experience in performing surveys and collecting specimens in the field. The leader managed overall logistics of each day's activity and supervised the workflow, providing troubleshooting as needed. Each team leader had a mobile phone and a satellite phone for use when outside of the cellular network, to communicate with each other and with the coverage team and health center.

##### Local health worker.

A health worker was selected by the local health office. This individual was fluent in the local languages and was already known to the community, including potential respondents. Under the direction of the team leader, the health worker assisted in field site setup, informed consent, phlebotomy, and locating participants who did not appear at the serosurvey gathering site.

##### Phlebotomist.

A phlebotomist for each serosurvey team was chosen from the Ethiopian Public Health Institute on the basis of pediatric phlebotomy experience, the most important criterion for this position. The phlebotomists were experienced in assuaging a parent's concerns during a blood draw, a potentially stressful situation.

##### Driver.

A driver was primarily responsible for maintenance of the vehicle and navigating to the blood collection sites in the field. However, the driver, who was usually fluent in the local language, also acted as a community liaison who interacted directly with participants and their families, including providing their transport, when possible, to and from the study gathering site.

##### Medical technologist.

One medical technologist worked at each field site, processing samples from both serosurvey teams and entering information from case report forms into a database. This individual had previous laboratory experience and was responsible for aliquoting the serum. When available, the medical technologist would also assist the serosurvey team in the field.

### Equipment.

**Figure 2.**
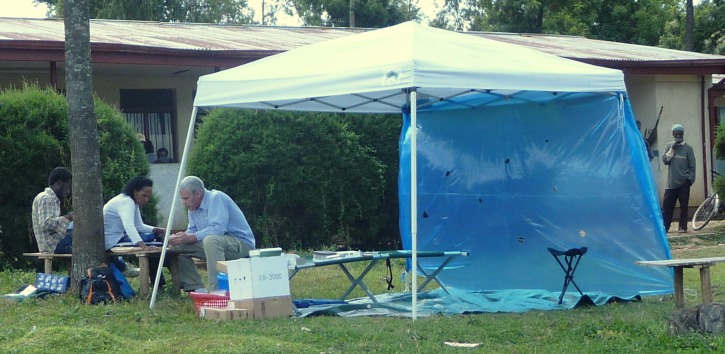
Field site setup. Each serosurvey team was equipped with a canopy, cot, tarp, and stools to create a sheltered space where informed consent and venipuncture could occur.

Reliable equipment with backup supplies allowed the serosurvey team to anticipate shortages and emergencies (Table 2). Most of these supplies were purchased in the United States and shipped to Ethiopia. Equipment was chosen to give each team the capacity to set up a sheltered field site with a canopy, cot, tarp, and stools where informed consent and venipuncture could occur (Figure 2

). Phlebotomy supplies included the option for venous blood draws with either a butterfly needle or needle and syringe combination, depending on the phlebotomist's preference. Two portable refrigeration units were set up at the local health facility, filled with frozen ice packs, and set to the lowest temperature setting. One unit was used for daily sample processing. The other unit was used for storage of aliquoted samples. This unit was opened as infrequently as possible, allowing its temperature to remain at or below −20°C. A portable generator was stored at the local health facility for use in the event of a power outage and failure of the facility's backup generator. A portable label maker gave each serosurvey team the capacity to prepare pre-printed adhesive labels in indelible ink for serum separator tubes and aliquot vials.

### Venipuncture.

**Figure 3.**
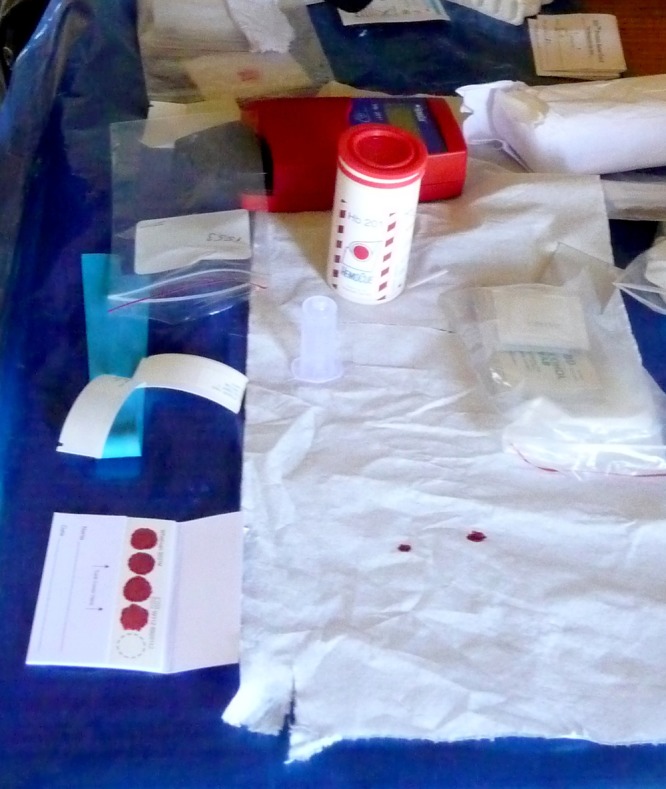
Hemoglobin measurement and filter paper collection. For each subject, 10 μL blood was used to fill a cuvette for point-of-care hemoglobin measurement with a HemoCue^®^ device, seen at top. Additional drops of blood were used to blot filter paper, completely filling each filter paper circle, seen at bottom.

A venous blood sample (maximum volume 3.5 mL) was drawn from each participant. Venipuncture was performed by an Ethiopian Public Health Institute (EPHI) phlebotomist with pediatric experience. The local health worker and team leader assisted with each blood draw, securing the child and assisting in the sample collection and processing. While papooses were available, the only tenable means for drawing blood from a child was found to be a parent holding the child with assistance from the serosurvey team. When a venous blood draw was unsuccessful, a fingerprick was obtained. Ten microliters of blood were used to fill a cuvette for point-of-care hemoglobin measurement. Additional drops of blood were used to blot filter paper, completely filling each filter paper circle, with up to five circles filled for each subject (Figure 3

). Each filter paper was labeled with the participant's serosurvey identification number and air dried for at least 4 hours, then placed in a sealable plastic bag with a desiccant pack. Sharps were placed in safety box containers that were subsequently disposed of at the local health facility.

### Serum collection and sample processing.

**Figure 4.**
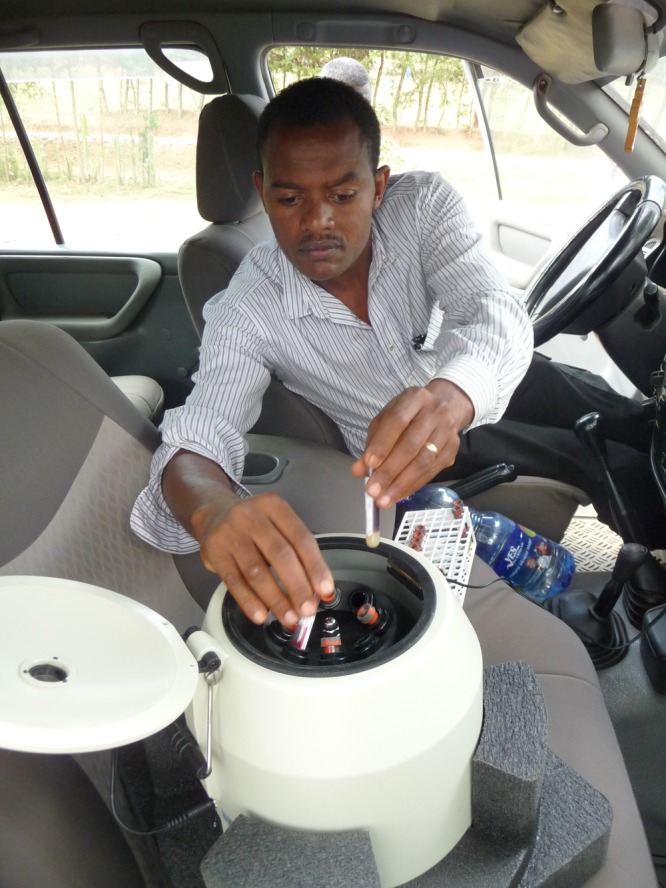
Serum samples were centrifuged on-site within 4 hours using a portable centrifuge (Portafuge^™^) that plugged into the field vehicle.

Venous blood was drawn either directly into a serum separator tube (SST) or into a syringe that was then used to fill an SST. When centrifuged, the SST assures that serum is physically separated from the clot by a gel layer. This means that when the centrifuged tubes are put into a refrigerated transport box, should cold hemolysis of erythrocytes occur (as happens in a proportion of refrigerated clot specimens), the serum remains separated from the hemolyzed erythrocyte fragments. One member of the serosurvey team centrifuged the serum separator tubes on-site within 4 hours using a portable centrifuge (Portafuge^™^) that plugged into the field vehicle (Figure 4

). The centrifuged SSTs were kept in an insulated container with frozen ice packs until further processing later that day.

Once or twice each day, the centrifuged SSTs in insulated containers with frozen ice packs were brought to a temporary laboratory set up at the local woreda health center. There, the medical technologist prepared aliquots of each subject's serum and transferred these aliquots to vials for frozen storage, following a standard operating procedure (SOP). Specimens yielded a maximum of 1.6 mL serum, from which four aliquots were prepared, with each vial containing approximately 0.4 mL serum (or less, depending on the actual volume of blood collected from the individual child). A pre-printed specimen identifier sticker on the serum separator tube bearing a code number was matched to each of the four vials. The tetrad of vials holding the aliquots of serum was then frozen and placed into a portable freezer before transport to EPHI in Addis Ababa. If the field site was within 1 day's drive to Addis Ababa, samples were transported by vehicle within the portable refrigeration unit, which was filled with frozen ice packs. Otherwise, samples were shipped by air courier to Addis Ababa to arrive on the same day, where they were picked up at the airport for storage at EPHI.

Two aliquots were shipped frozen to the Center for Vaccine Development (CVD) Applied Immunology Section Laboratory in Baltimore for definitive testing for antibodies against selected vaccine antigens and for preliminary training of Ethiopian serologists. The remaining two aliquots of serum were stored at EPHI, where they were used for additional serologic training and serological testing, with the CVD serving as the reference laboratory.

Filter papers were stored at room temperature in a secure, dry location at the temporary laboratory at the woreda health center until they could be transported to EPHI in Addis Ababa. They were then sent to the CVD Applied Immunology Section Laboratory in Baltimore for analysis.

### Data collection.

Coverage survey enumerators recorded the woreda and district, enumeration code, household list number, selection number, and GPS location for each participant. Participants also received a unique serosurvey identification number. The serosurvey team recorded data manually on a case report form. The health center technician entered these data into an Epi Info 7 database using a Netbook. This database was queried to produce weekly progress reports.

## Results

The immunization coverage survey team collected data on 1,181 children across all three woredas; of these, 1,023 (87%) enrolled in the serosurvey (Table 3). Impressively, 81–90% of children enrolled in the coverage survey in each woreda also participated in the serosurvey. The duration for completion of the coverage survey and serosurvey among ∼400 chidren in each woreda ranged from 12 days in Hintalo Wajerate to 20 days in Arbegona. Serum was successfully collected from 96–97% of serosurvey enrollees in each woreda, with no adverse events related to these blood draws. Enough serum was collected from each participant for between 2.1 and 2.6 serum aliquots.

Several strategies contributed to this high level of participation, efficiency, and successful sample collection, as described below.

### Coordination between the coverage survey and serosurvey teams.

**Figure 5.**
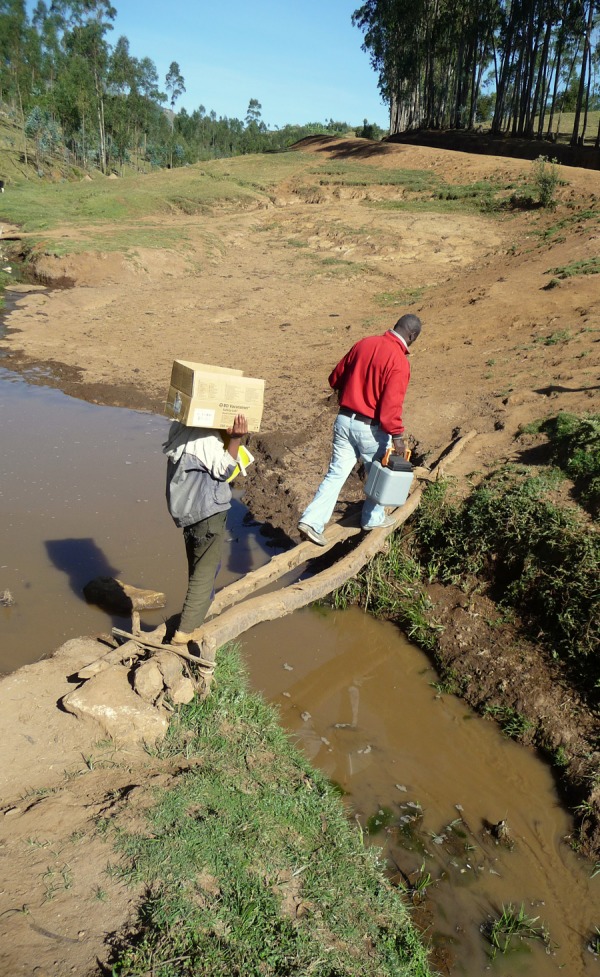
When temporary workstations were inaccessible to vehicles, supplies and samples were carried on foot.

The serosurvey teams set up temporary workstations in school and church compounds, health posts, and, on rare occasions where no shelter was available, under the shade of trees. Supplies and samples were carried on foot to and from the vehicle when terrain precluded vehicle access to the workstation (Figure 5

). At the time of the administration of the coverage survey questionnaire, the coverage survey team informed potential serosurvey participants to meet the serosurvey team at these workstations at a specified time. Once the caregiver arrived at the serosurvey site, the referral slip was verified and the caregivers listened to an audio recording of the consent form in the local language, which provided information about the rationale for collecting blood from the child, the precise procedures, and potential risks and benefits. After listening to the audio recording, each caregiver was given an opportunity to have any questions answered and they were then asked to sign the consent form. If a caregiver was illiterate and unable to sign the form, they were asked to “sign” with a thumb print after pressing their thumb on an ink pad.

Each evening, the coverage survey team and serosurvey teams met to discuss logistics and planning. Representatives from the local health office joined these discussions. Topics discussed included the pace of each team's efforts to maximize coordination between the two groups; timing and locations of serosurvey visits; accessibility of sites, and incorporation of input and feedback from the local health office. Using a detailed woreda map, three to four clusters were grouped into zones and movement plans were jointly drawn up by coverage survey supervisors, serosurvey team leaders, and participating woreda health officers.

Each morning, the coverage survey and serosurvey team leaders verified the day's activities, including the serosurvey gathering site and time and the logistics for travel. They also reviewed information collected for the previous day's participants and resolved any discrepancies in data between the coverage survey and serosurvey. With the aid of a checklist, each serosurvey team assembled equipment and supplies for the day's activities.

### Medical evaluation and treatment, including anemia.

Caregivers were questioned about their child's health, including previous intestinal parasitic infections, nutrition, and any specific health concerns they had. A drop of the collected blood samples was placed in a cuvette and used to measure the hemoglobin level of participants (HemoCue^®^).

The presence and severity of anemia was determined using 2011 Ethiopia Demographic and Health Survey^3^ thresholds modified in consultation with the Ethiopian Pediatric Society and scientific and ethical review committee of the Ethiopian Public Health Institute (Table 4). Every child with anemia was given iron. Anemic toddlers and any children suspected of having intestinal parasitic diseases received a broad spectrum oral anthelminthic agent (mebendazole). All anemic children were referred to the health center for follow-up.

Vitamin A supplementation was provided to any child who had not received supplementation in the past month, per the caregiver.

Serosurvey teams were equipped with medications to treat common conditions such as scabies and dehydration. Treatment was provided without charge when these conditions were suspected.

### Community buy-in.

Enrollment exceeded 80% of coverage survey participants in each of the three woredas. Sensitization of the local population to the project by community leaders was integral to achieving community acceptance and facilitated buy-in at each site. UI-FHS spent several weeks discussing the project with religious and community leaders as well as health officials to sensitize communities to the surveys before their start. Gaining the support of religious leaders, particularly with respect to blood sampling, was also important in securing the support of community leaders. Understanding and considering the cultural and religious needs of the community in advance was important for successfully engaging the target community on time. This involved consideration of the market days for rural communities, saint day celebrations, church attendance, and semi-nomadic community seasonal mobility patterns in planning the dates for the coverage and serosurveys.

Local health workers who were already known to the families of participants described the study objectives in the local language. In consultation with the serosurvey team leaders, the local health workers responded to the questions and concerns of caregivers. Community embrace of the serosurvey was also strengthened by the benefits available to serosurvey participants, including detection and treatment of anemia, vitamin A supplementation, and evaluation of select medical conditions. Serosurvey team leaders provided medical evaluation of any children in the community with ailments, when requested by the child's parents or other caregiver.

## Discussion

We successfully integrated serosurveys with immunization coverage surveys in three remote regions of Ethiopia in 2013. This effort posed serious logistical challenges that were overcome by applying several broad strategies and implementing specific tactics. Examples include: the serosurvey team assigned clearly defined primary and backup roles to each team member, needed equipment and supplies were anticipated and chosen with great care, steps were taken to continually maintain close coordination and communication between the coverage survey and serosurvey teams, and efforts were undertaken to ensure community buy-in at each field site, including by providing point-of-care measurement of hemoglobin levels to detect and treat anemia and to diagnose and treat other common pediatric ailments upon the request of the child's parent or caregiver.

Whereas serosurveys have been successfully conducted in developing countries with pediatric immunization schedules that follow EPI guidelines,^5^,^6^ little information exists on how to overcome the substantial logistical challenges that such efforts entail, particularly in rural regions. Importantly, the strategies for equipment and supply selection, team composition, and community buy-in described here have widespread application for the performance of serosurveys for any reason in isolated developing country settings.

Tactics that should be used in future serosurveys include the assignment of a single survey code number for both the immunization coverage survey and the serosurvey to facilitate harmonization of coverage survey and serosurvey databases. Additional efforts to synchronize data collection forms and databases between the coverage survey and serosurvey before initiation will also expedite data analysis.

Efficient workflow for the serosurvey was highly dependent on the competence of the phlebotomists. Thus, prior pediatric phlebotomy experience involving infants and toddlers was critical to their success and should be emphasized as the most important selection criterion when choosing a phlebotomist.

One potential limitation of these results is that some of the challenges encountered may be specific to this study. Future serosurveys at other sites may encounter additional local challenges not anticipated in this study, such as freezing ambient temperatures, rather than the elevated temperatures we dealt with in Ethiopia. Freezing temperatures would pose distinct challenges to sample and cold chain maintenance not mentioned in this article. A serosurvey may also face fewer challenges than those described here. For example, each of the Ethiopian field sites in this project had a different primary language, and consent forms and audio cassettes had to be translated to address these differences. If teams work in multiple sites where there is only one spoken language, audio cassette translations may be unnecessary, albeit still helpful in regions with low literacy.

The WHO immunization coverage cluster survey reference manual has provided guidelines for successful execution of a coverage survey.^1^ The serosurvey equipment and supplies, team composition, and close coordination with a coverage survey were critical to successfully performing a serosurvey in concert with a coverage survey. The strategies and tactics described here will be useful for future serosurvey planning and management.

## Figures and Tables

**Table 1:** Responsibilities of serosurvey team members

Serosurvey team members	Responsibilities
Team leader	
		Supervision, logistics, and workflow
		Collection of data for each subject's CRF
		Interpretation of the child's Hb level using the HemoCue^®^ Hb 201 + Analyzer
		Centrifuge blood so that serum can be separated
		Oversight and quality of database
		Backup phlebotomist
		Backup for preparing DBS on filter paper
		Backup for measurement of the child's Hb level using the HemoCue Hb 201 + Analyzer
Backup for deworming treatment and iron supplementation
Deputy team leader (when available)	
		Supervision, logistics, and workflow as needed
		Collection of data for each subject's CRF
		Backup phlebotomist
		Backup for preparing DBS
		Backup for Hb measurement and treatment
Phlebotomist	
		Collect and label serum in SST
		Measure the child's Hb level using the HemoCue Hb 201 + Analyzer
		Centrifuge blood so that serum can be separated
		Filter paper preparation
		Maintain a log of all clinical specimens obtained from the subjects
		Backup to set up the worksite
		Backup to obtain informed consent from the child's parent or caretaker
Local health worker	
		Translation and communication services in local languages
		Obtain informed consent from the child's parent or caretaker
		Assist holding child during phlebotomy and comforting parents and child
		Primary dispenser for deworming treatment and iron supplementation
		Backup phlebotomist (if trained and experienced in collecting blood from young children)
		Backup for preparing DBS on filter paper
Driver	
		Transportation for the team members and for moving clinical specimens to intermediate cold or frozen storage
		Assist in communicating in the local language(s) with parents, caretakers and others in the household and community
		Assist in setting up the work site
		Backup for centrifuging blood specimens to separate and collect serum
		Transportation of samples for long-term storage to EPHI in Addis Ababa
Health center technician (one per woreda)	
		Organize the collection of clinical specimens
		Prepare logs for specimen collection
		Data entry and database maintenance
		Store clinical specimens and arrange for shipping

CRF = case report form; DBS = dried blood spots; EPHI = Ethiopian Public Health Institute; Hb = hemoglobin; SST = serum separator tube.

**Table 2:** Serosurvey equipment and supplies, including quantity and brand, when informative

Equipment	Quantity	Brand
Setup		
8 × 10 ft tarpauline	2	Kotap Heavy Duty Tarp (Kotap America Ltd., Lawrence, NY)
10 × 10 ft canopy	2	E-Z Up Canopy (E-Z UP, Inc., Norco, CA)
Folding camp cot	3	Texsport Deluxe Folding Camp Cot (Texsport, Inc., Houston, TX)
Collapsible stools	3	Rothco Black Collapsible Stools (Rothco, Ronkonkoma, NY)
Cassette player	2	Coby CXCD248 Portable Stereo Cassette Player (Coby Electronics Co., Ltd., Guangzhou, Guangdong, China)
D batteries for cassette player	2 boxes	Energizer Industrial Alkaline batteries (Energizer, St. Louis, MO)
Audio cassettes (90 minutes)	6	Maxell Dictation and Audio Cassettes (Maxell, Woodland Park, NJ)
Hemoglobin-measuring device	4	HemoCue^®^ HB201 + Analyzer (HemoCue America, Brea, CA)
Hemoglobin microcuvettes	10 boxes of 200	HemoCue Hb 201 Hemoglobin microcuvettes
Generator (3000 W; 120V/240V; 60 Hz; unleaded gasoline; portable)	1	Newstar 3000 Generator (New Star Technology, Inc., Montclair, CA)
Satellite phones	4	Iridium 9555 (Iridium Communications, Inc., McLean, VA)
GPS	3	Garmin eTrex Venture HC GPS Receiver (Garmin International, Inc., Olathe, KS)
Netbook for data entry	1	ASUS Eee PC (Asus Computer International, Fremont, CA)
Phlebotomy		
Tourniquets	2 cases of 500 each	Fisherbrand Nonlatex Disposable Tourniquet (Fisher Scientific, Pittsburgh, PA)
5 mL syringes	10 pack of 50 each	BD Safety-Lok Syringes (Becton, Dickinson and Company, Franklin Lakes, NJ)
Labels	40 sets	Brady LABXPERT High-Performance Laboratory Polyester Labels (Brady Corporation, Milwaukee, WI)
Label maker	2	Brady LABXPERT v2.0 Labeling System
AA batteries for label makers	2 boxes of 24	Energizer Industrial Alkaline batteries
Vacutainer holders	2 cases of 1000 each	BD Vacutainer Tube Holder
23-gauge butterfly needles, 22-gauge needles	8 cases of 200 butterfly needles, 1 pack of 10 22-gauge boxes	BD Vacutainer Safety-Lok Blood Collection Sets, BD PrecisionGlide Needles
Alcohol swabs	2 cases of 1200 each	BD Brand Isopropyl Alcohol Swabs
Gauze sponges, 2 × 2 in	2 cases of 25 packs each	Fisherbrand Gauze Sponges
Gauze sponges, 4 × 4 in	1 case of 25 packs of 200 each	Fisherbrand Gauze Sponges
Bandages (3/4 × 3 in strips)	2 cases of 12 packs each	BSN Jobst Coverlet Adhesive Dressings (BSN medical Inc., Charlotte, NC)
Lancets (200/box)	4 boxes	BD Microtainer Contact-Activated Lancet.
Serum separator tubes (3.5 mL)	2 cases of 10 packs of 100 each	BD Vacutainer Venous Blood Collection Tubes: SST, 3.5 mL
Safety box containers for used sharps disposal	1 case of 24 containers	BD Multi-Use Nestable Sharps Collectors, 8 quart
Papoose boards	5	Olympia Medical 50510 Small Papoose Boards (Natus Medical Inc., Pleasanton, CA)
Gloves (latex, nitrile, or equivalent)	Small: 6 pack of 100 each	Fisherbrand Powder-Free Latex Exam Gloves, Kimberly-Clark Professional Purple Nitrile Exam Gloves (Kimberly-Clark Corporation, Irving, TX)
	Medium: 2 cases of 10 packs of 100 each	
	Large: 10 pack of 100 each	
Filter papers	35 packs of 100 each	Whatman 903 Protein Saver cards (GE Healthcare Bio-Sciences, Pittsburgh, PA)
Sealable plastic bags	35 packs of 100 each	Whatman plastic sample bags
Desiccant packs	35 packs of 100 each	Whatman 903 desiccant packs
Paper towels	–	
Biohazard bags	1 pack of 200	Fisherbrand Polypropylene Biohazard Autoclave Bags, 24 × 36 in.
Biohazard bag holders	2	Bel-Art Scienceware Clavies Biohazard Bag Holders, 24 × 36 in. (Bel-Art Products, Wayne, NJ)
Treatment		
Iron supplement	1 bottle per participant	HaemUp^™^ iron syrup with folate (available locally) (Cadila Pharmaceuticals PLC, Addis Ababa, Oromia, Ethiopia)
Mebendazole	1 treatment per participant	Available locally
Vitamin A (100,000 IU capsules)	1 capsule for infants	Available locally
	2 capsules for toddlers	
Serum processing		
Test tube racks		Available locally
Centrifuge	5	Portafuge^™^, Model E8-3000 (LW Scientific, Lawrenceville, GA)
Insulated container for serum samples	2	Available locally
Cold packs	Multiple	Available locally
Portable refrigeration unit	5	Engel portable fridge/freezer, Model no. MT45F-U1 (Engel Machinery Inc., York, PA)
Screw cap cryovial 2-mL microcentrifuge tubes	3 cases of 5 packs	Screw Cap Micro Tubes, 2 mL (Sarstedt AG & Co., Nümbrecht, Germany)
100–1000 μL Pipettors and pipette tips	2 pipettors	Finnpipette F1 pipettors (Thermo Fisher Scientific Inc., Waltham, MA)
	3 cases of 960 pipette tips	Finntip filtered pipette tips (100–1000 μL) (Thermo Fisher Scientific Inc., Waltham, MA)
Tape		Strate-Line Autoclavable Tape, locally available clear tape (Propper Manufacturing, Long Island City, NY)
Freezer storage boxes with separators	9 packs of 12 boxes each	Thermo Scientific Freezer Fiberboard Storage Boxes and Box Dividers (Thermo Fisher Scientific Inc., Waltham, MA)
Bleach solution, 10%	–	Available locally
Paperwork		
Protocols	–	–
Informed consent forms	2 per participant	–
Case report forms	1 per participant	–
Serum separator tube sample log sheet	–	–
Filter paper sample log sheets	–	–
Hemoglobin measurement log sheets	–	–
Serum specimen log sheet	–	–
Paper pads	12	Universal Legal Ruled Paper Pads (United Stationers Supply Company, Deerfield, IL)
Ink pads	2 per team	Available locally
Black pens	6 dozen	Paper Mate ComfortMate Grip Retractable Ballpoint Pens (Paper Mate Products, Inc., Oak Brook, IL)
Black permanent markers	24	Sharpie Ultra Fine Tip Permanent Markers (Newell Rubbermaid, Atlanta, GA)
Scissors (5′′)	4	Sparco Pointed Tip Grip Bent Handle Scissors (S.P. Richards Company, Smyrna, GA)
Storage clipboard	5	OIC Slim Storage Clipboard (Officemate International Corporation, Edison, NJ)

GPS = global positioning system; SST = serum separator tube.

**Table 3:** Coverage survey and serosurvey enrollment for each woreda

Woreda (region)	Survey duration	Enrollment		Successful serum collection	Mean aliquots per participant
		Coverage survey	Serosurvey		
Arbegona (SNNPR)	20 days	395	350 (89%)	338/350 (97%)	2.6
Assaieta (Afar)	15 days	390	317 (81%)	303/317 (96%)	2.1
Hintalo Wajerate (Tigray)	12 days	396	356 (90%)	341/356 (96%)	2.4
All woredas	47 days	1,181	1,023 (87%)	982/1,023 (96%)	2.4

SNNPR = Southern Nations, Nationalities, and Peoples' Region.

**Table 4:** Anemia evaluation and treatment guidelines (based on 2011 Ethiopia Demographic and Health Survey^3^ thresholds modified in consultation with the Ethiopian Pediatric Society and scientific and ethical review committee of the Ethiopian Public Health Institute) and vitamin A supplementation guidelines^4^

Therapeutic interventions for different levels of anemia				
Severity of anemia defined by Hb level (g/dL)	Iron supplement (HaemUp^™^ iron syrup with folate)	Antihelminthic (mebendazole)	Vitamin A (100,000 IU for infants and 200,000 IU for toddlers^4^)	Parent should bring the child to the nearest health center
Mild anemia (Hb 10.0–11.9 g/dL)	Yes	Toddlers only	Yes	In 14 days
Moderate anemia (Hb 5.0–9.9 g/dL)	Yes	Toddlers only	Yes	Within the next few days
Severe anemia (Hb < 5.0 g/dL)	Yes	Toddlers only	Yes	Same or next day

Hb = hemoglobin.
